# An optimal scheduling method of integrated energy system in industrial parks considering carbon trading and electric vehicle charging load

**DOI:** 10.1038/s41598-025-24014-7

**Published:** 2025-11-20

**Authors:** Hu Wendong, Shen Hao, Ma Mingyu, Tian Zhijie, Dong Yu, Zhang Bo, Xie Wei

**Affiliations:** 1 State Grid Hebei Electric Power Co., Ltd. Handan Power Supply Branch, Handan, 056002 Hebei China; 2 State Grid Xiong’an Sgitg Digital Technology Co., Ltd., Xiong’an New Area, 071700 Hebei China

**Keywords:** Carbon trading, Electric vehicles, Integrated energy system, Scheduling strategy, Industrial park load, CPLEX, Fuel cells, Renewable energy

## Abstract

In order to achieve an objective of carbon peaking and carbon neutrality and optimize the multi-energy utilization in industrial parks, an optimal scheduling method of integrated energy system for industrial parks is proposed. Firstly, a comprehensive energy system of industrial parks is designed based on the characteristics of energy diversification, which gathers electricity, heat, and hydrogen energy in industrial parks. Then, using electric vehicles as flexible and adjustable loads to achieve the scheduling process to solve the problem of weakly load adjustability. Considering the carbon trading mechanism and the EV charging load adjustment ratio, an economic mathematical model with the objective function of minimum system total operating cost is established. Finally, the CPLEX solver is transferred by MATLAB to comparative and analyze four schemes. It shows that the carbon emission by 4.5% in daily operation with the proposed scheduling strategy, and the operating costs can be reduced by 1.54% when the load ratio of EV is adjusted below 30%. It can be seen that the cost is reduced, which provides a effective method for the optimal scheduling of energy systems in industrial parks.

## Introduction

The gradual depletion of fossil energy has led to the intensification of energy worldwide Scarcity. The issue of carbon emissions from fossil fuels has also received increasing attention. On September 22, 2020, China’s “dual car-bon” goals were proposed at the 75th United Nations General Assembly. Industrial production activities in the industrial park are one of the sources of carbon emissions. Building a comprehensive energy system for the industrial park, integrating clean and pollution-free renewable energy such as the integrated wind-solar, reducing the cost of power grid purchase, and reducing carbon emissions are currently very promising development directions. For the economic optimization of the integrated energy system, the current research has done relatively com-prehensive research on the energy supply side and the demand side respectively. The characteristics of energy sup-ply and storage equipment and their impact on economic benefits have been considered^1^. The environmental benefits of grid connection of renewable energy power generation and system operation and maintenance costs have been considered^2^.Their demand response model has been used to achieve the goal of efficient utilization of energy and improving the overall benefits of both supply and demand sides by shifting the time scale of schedulable loads^3–5^. However, there are still relatively few studies on integrated energy system aiming at the characteristics of industrial parks. The biggest difference between the industrial park and the general scenario is its load charac-teristics. Most of the loads are used for industrial production. It has non-adjustable loads with low volatility and strong regularity, and there are few flexible loads that can be adjusted flexibly. The daytime load in the park is higher than the nighttime load, the daytime load is relatively stable^6^, and the regulation is poor, which is not applicable to the model proposed for the traditional energy system.

One of the prominent features of the comprehensive energy system in industrial parks is the diversity of energy resources. There are many kinds of energy needs in the system, such as electricity, cold, hot and gas. The optimal dispatch of multi-energy equipment and the rational transformation of different energy resources can greatly improve the economy and operation efficiency of the system. The application of hydrogen energy in the integrated energy system can be divided into two ways.

When performing unified scheduling as hydrogen load, electrical load, and heat load, a comprehensive energy system for hydrogen and electricity has been established^7,8^, using renew-able energy to electrolyze water to produce hydrogen to supply hydrogen loads. An economic optimization scheduling model established considering operating costs, environmental costs, and the use of hydrogen to supply hydrogen loads and methanation to supply gas turbines to achieve efficient energy utilization can improve the economy and low-carbon performance of the integrated energy system^9,10^. The other is as energy storage. Having considered that hydrogen energy storage is only used for power storage with low efficiency, hydrogen energy storage is usually used as a source of multiple energy cogeneration to complete the conversion of electricity, hydrogen, and other energy forms. For example, a hybrid energy storage system combining hydrogen storage and heat storage has been studied to meet both electricity and heat supply^11^, and a scenario based algorithm has been developed to solve the uncertainty in renewable energy supply and demand; It has been used as the heat engine of the combined cooling, heating and power system^12–14^, and an economic scheduling model with the objective function of minimizing the operation cost of the day ahead scheduling has been constructed^15^, a multi-objective stochastic optimum energy management model for a renewable-supported hydrogen-based community is proposed with mixed-integer linear programming method^16^. Chaos enhanced fireworks algorithm has been used to optimize the solution. A stochastic optimal scheduling strategy for an electricity hydrogen gas heat integrated energy system based on improved spectral clustering method has been proposed^17^, having taken into account the uncertainty of source and charge, which is conducive to the flexible utilization of hydrogen energy. It is necessary to integrate the demand for industrial hydrogen and the characteristics of large dispatching energy in the park for the use scenarios of hydrogen energy. The utilization of hydrogen energy is targeted at high efficiency, low cost, and pollution-free based on the environmental advantages of high efficiency, large energy storage capacity, and clean and pollution-free hydrogen energy storage and multi-energy cogeneration.

In the context of the dual carbon goal of “carbon peak, carbon neutral”, some research on integrated energy systems actively have explored new paths, such as the introduction of carbon trading mechanisms in optimal scheduling^18–21^, adding carbon trading costs to costs, and reducing carbon emissions through the system’s opti-mal scheduling process. An optimal scheduling model considering carbon capture^22^, utilization, and storage has been established to reduce the carbon emissions of the electricity gas heat integrated energy system. The example in the paper proves that the proposed model reduces the carbon emissions by 36.79%.

In recent years, the number of electric vehicles has grown rapidly. Adding the charging process of electric vehicles to the scheduling process of the power grid has become one of the key issues of electric vehicles^23,24^, so as to alleviate the peak valley characteristics of the power grid load. At the same time, the utilization rate of renewable energy and economic benefits can be improved by optimizing the load curve of electric vehicles. The scheduling of electric vehicle charging stations has been studied to achieve the goal of improving economy and enhancing renewable energy consumption through optimizing the guidance mechanism ^25–27^. However, these studies on optimal scheduling of electric vehicles in charging stations are all aimed at charging users, with strong randomness in charging behavior. Therefore, it is necessary to schedule electric vehicles through a guidance mechanism. Electric vehicles in industrial parks are mainly used for goods transportation, employee commuting, etc. Electric vehicles have strong regularity in working hours, and their charging behavior during non- working hours is relatively flexible. Therefore, based on internal sources, load balance, and economic considerations, the system can reallocate some of the charging loads in a timely manner while ensuring that the total charging load within a day is met, thereby taking the electric vehicle charging load as an adjustable load, assisting the system in peak shaving and valley filling, and improving comprehensive benefits.

It aims to achieve peak shaving and valley filling in the large power grid, improve the energy efficiency of hy-drogen storage, and increase system revenue. An integrated energy system containing electricity, heat and hydro-gen was established to carry out day ahead optimal scheduling, which introduces a stepped Carbon emission trading mechanism, and includes the cost of Carbon emission trading to reduce carbon emissions in the process of optimal scheduling. The electric vehicle charging load is regarded as an adjustable load based on the industrial load characteristics and electric vehicle charging characteristics within the industrial park, which is considering the guidance of charging time of use electricity price, and adjusting the electric vehicle load curve from a system perspective to promote the consumption of renewable energy. The system operation optimization scheduling model is established, which takes the lowest total operating cost as the goal, considers the constraints such as equipment operating power, electric vehicle regulation degree. The CPLEX solver is used to obtain waveform curve, the day ahead scheduling results under different scenarios can be compared. The results show that the carbon emissions can be significantly reduced by the carbon emission trading system, the consumption of renewable energy can be promoted by the charging load curve of electric vehicles, reduce, the overall economy of the system was improved and the charging costs were reduced.

## Integrated energy system

### Integrated energy system structure

**Fig. 1:**
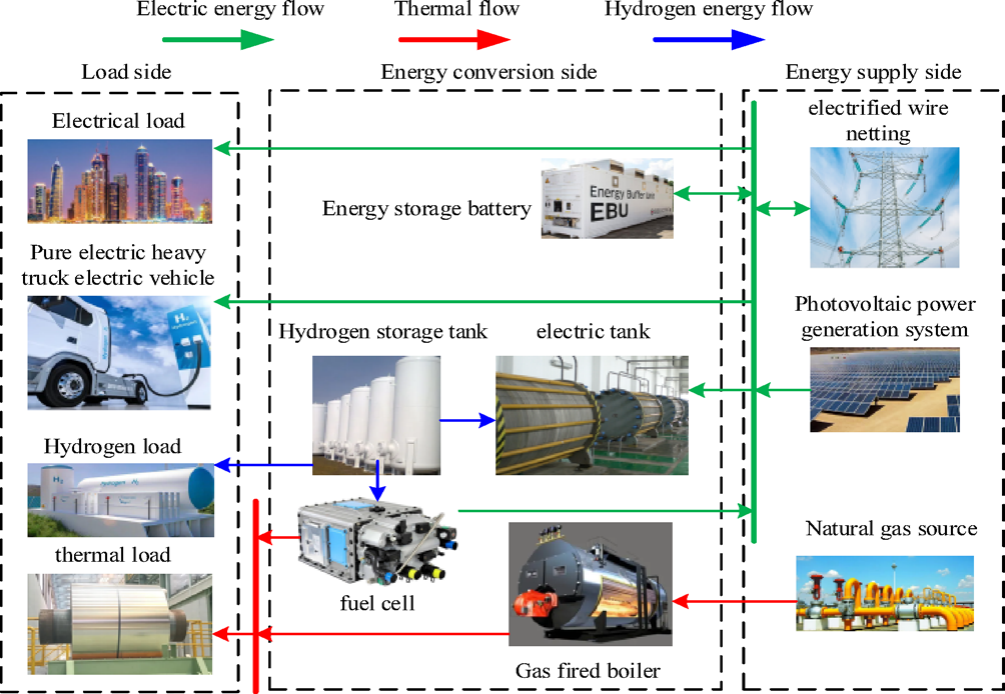
Schematic diagram of integrated energy system in industrial park.

The integrated energy system constructed in this paper includes photovoltaic power generation system, battery, electrolytic cell, hydrogen storage tank, fuel cell cogeneration equipment, gas boiler, electricity, heat and hydrogen load. The electric load includes the adjustable electric vehicle load and the basic electric load in the industrial park. When the system power is insufficient or the photovoltaic power generation still has surplus power, it can interact with the power grid^28^. Fuel cell power generation is accompanied by heat generation. This part of heat energy is recycled to supply the heat load required by some residential hot water, and the insufficient heat load is provided by gas boiler. Such as Hydrogenation stations, chemical plants, etc., in the industrial park need hydrogen as raw material, so the daily supply of hydrogen in the integrated energy system is required to be no less than a certain value. The structure of integrated energy system is shown in Fig. 1.

### Electric vehicle

Electric vehicles in the industrial park are mainly divided into three types: electric commuter vehicles, private electric vehicles and electric business vehicles. Among them, the electric commuter bus operates in shifts, mainly for employees in the park to commute and travel in the park. Private electric vehicles in the industrial park are mainly used by people working and living in the park. In addition to commuting, there are also personal travel arrangements, but the charging time is relatively free and has a certain flexibility. Business vehicles are used by enterprises in the park to carry out specific travel tasks^29^. The charging time is usually once a day after the end of the trip, because the use time is less, the dispatchable range is the largest. A stochastic charging load prediction model for electric vehicles has been established through Monte Carlo prediction to obtain the total charging load of electric vehicles in an industrial park for a day^30^. When the electric vehicle is charged in disorder, the electric vehicle is added to the electric load of the industrial park as a random electric load, regardless of the charging price, and the load is predicted according to the charging habit of the electric vehicle; After the electric vehicle is optimized, the economic guidance of the time-of-use electricity price is considered, and the load is adjusted through the system dispatching. Because the electric vehicle load is optimized as the internal load of the industrial park, the electric vehicle charging cost is included in the total operating cost of the integrated energy system and it will not be calculated separately.

### Electric vehicle charging model considering time-of-use tariff guidance

After the electric vehicle is added to the optimization as the load of the industrial park, based on the guidance of the time-of-use electricity price, under the premise that the total charging demand remains unchanged, through the optimization scheduling, a part of the electric vehicle load will be moved from the peak section to the flat and valley section and the power purchase cost of the system will be reduced in the peak section.

1. Charge demand constraints (the total charge before and after optimization remains unchanged)
  1 $$\begin{array}{*{20}c} {\mathop \sum \limits_{t = 1}^{T} P_{{{\text{EVB}}}} \left( t \right) = \mathop \sum \limits_{t = 1}^{T} P_{{{\text{EVA}}}} \left( t \right)} \\ \end{array}$$
  where, ${\text{P}}_{{{\text{EVB}}}} \left( {\text{t}} \right)$ and ${\text{ P}}_{{{\text{EVA}}}} \left( {\text{t}} \right)$ are respectively the charging power of electric vehicle load at time t after random charging and time-of-use tariff guidance. (1) Manual adjustment and sensitivity analysis, manually changing the values of one or more parameters, re solving the model, and observing the changes in the optimal solution and optimal value. (2) Automatic Calibration/Optimization, which constructs parameter adjustment itself as an optimization problem, i.e. an “optimization problem of the optimization model”, involves meta modeling or machine learning. (3) Stop when the loss function converges or reaches the number of iterations.
2. Adjustable proportional constraint of electric vehicle charging load
  2 $$\frac{{\mathop \sum \nolimits_{t = 1}^{T} \left| {P_{{{\text{EVB}}}} \left( t \right) - P_{{{\text{EVA}}}} \left( t \right)} \right|}}{{\mathop \sum \nolimits_{t = 1}^{T} P_{{{\text{EVB}}}} \left( t \right)}} \le \delta_{max}$$
  where, ${\updelta }_{{{\text{max}}}}$ is the upper limit of the adjustable proportion of electric vehicle charging load, which is related to the type and quantity proportion of electric vehicles in the industrial park. A large number of EV charging behaviors are identified to find the optimal charging and discharging scheduling scheme while satisfying all physical and user constraints by mathematical programming, in order to achieve system level goals such as minimizing total costs, smoothing load curves, and maximizing renewable energy consumption.

## Optimal scheduling model of integrated energy system

The optimal scheduling model of integrated energy system includes objective function and constraint condi-tions.

### Objective function

The objective function to minimize the total cost of daily operation is:

3 $$\begin{array}{*{20}c} {F = \min \left( {f_{{{\text{op}}}} + f_{{{\text{sta}}}} + f_{{{\text{grid}}}} + f_{{{\text{buy}}}} + f_{{{\text{exp}}}} } \right) } \\ \end{array}$$

#### ${ f}_{\text{op}}$ is the operation and maintenance cost of equipment in the integrated energy system

4 $$f_{op} = k_{pv} P_{pv} + k_{{{\text{bat}}}} P_{{{\text{bat}}}} + k_{{{\text{el}}}} P_{{{\text{el}}}} + k_{{{\text{fc}}}} P_{{{\text{fc}}}} + k_{{{\text{gb}}}} P_{{{\text{gb}}}}$$

where, $k_{{{\text{pv}}}}$, $k_{{{\text{bat}}}}$, $k_{{{\text{el}}}}$, $k_{{{\text{fc}}}}$ and $k_{{{\text{gb}}}}$ are respectively the operation and maintenance costs per unit power of photovoltaic power generation system, battery, electrolytic cell, fuel cell and gas boiler; $p_{{{\text{pv}}}}$, $p_{{{\text{bat}}}}$, $p_{{{\text{el}}}}$, $p_{{{\text{fc}}}}$ and $p_{{{\text{gb}}}}$ are respectively the electrical power, battery power, electrolytic cell power, fuel cell power and gas boiler power generated by the photovoltaic power generation system.

#### $f_{{{\text{sta}}}}$ is the start and stop cost of equipment in the integrated energy system

5 $$\begin{array}{*{20}c} {f_{{{\text{sta}}}} = f_{{{\text{batsta}}}} + f_{{{\text{elsta}}}} + f_{{{\text{fcsta}}}} + f_{{{\text{gbsta}}}} } \\ \end{array}$$

where, $f_{{{\text{batsta}}}}$, $f_{{{\text{elsta}}}}$, $f_{{{\text{fcsta}}}}$, $f_{{{\text{gbsta}}}}$ are the start-up costs of battery, electrolytic cell, fuel cell and gas boiler respectively.

#### $f_{{{\text{grid}}}}$ is the cost of electric energy interaction with the grid

6 $$f_{{{\text{grid}}}} = C_{{{\text{buyE}}}} \times \mathop \sum \limits_{{{\text{t}} = 1}}^{{\text{T}}} P_{{{\text{fromgrid}}}} \left( t \right) - C_{{{\text{pv}}}} \times \mathop \sum \limits_{{{\text{t}} = 1}}^{{\text{T}}} P_{{{\text{togrid}}}} \left( t \right)$$

where, $C_{{{\text{buyE}}}} { }$ is the time-of-use electricity price of the grid; $P_{{{\text{fromgrid}}}}$ is the power purchased from the grid at t moment; $C_{{{\text{pv}}}}$ is the photovoltaic grid price; $P_{{{\text{togrid}}}}$ is the power of PV grid at the moment of t.

#### $f_{{{\text{buy}}}}$ is the cost of purchasing natural gas for the integrated energy system

7 $$\begin{array}{*{20}c} {f_{{{\text{buy}}}} = C_{{{\text{gas}}}} \times \mathop \sum \limits_{{{\text{t}} = 1}}^{{\text{T}}} V_{{{\text{gas}}}} \left( t \right)} \\ \end{array}$$

where, $C_{{{\text{gas}}}}$ is the unit price of natural gas; $V_{{\text{gas }}} \left( t \right)$ is the volume of natural gas purchased at the moment of t.

#### ${ }f_{{{\text{exp}}}}$ is the equivalent environmental cost

8 $$\begin{array}{*{20}c} {f_{{{\text{exp}}}} = f_{{{\text{exp}}1}} + f_{{{\text{exp}}2}} } \\ \end{array}$$

9 $$f_{{{\text{exp}}2}} = \mathop \sum \limits_{{{\text{t}} = 1}}^{{\text{T}}} \sum\limits_{i = 1}^{{\text{I}}} {\delta_{i} Ce_{i} \left( {P_{{{\text{gb}}}} \left( t \right) + P_{{{\text{fromgrid}}}} \left( t \right)} \right) }$$

where, ${f}_{\text{exp}1}$ is the carbon transaction cost; ${f}_{\text{exp}2}$ is the cost of pollutant emission generated by electricity and gas purchase; ${\delta }_{i}$ is the cost of pollutant treatment; ${Ce}_{i}$ is the pollutant emission per unit output; $I$ is the type of pollutant^31^. (1) The MILP model typically assumes that carbon prices are exogenously given (i.e., a fixed parameter or several deterministic scenarios), which is seriously unrealistic. (2) The ability to handle uncertainty is limited, and the carbon trading market is full of uncertainty, including price fluctuations, adjustments to quota allocation methods, and the impact of technological progress on emission reduction costs. (3) Carbon trading in models is often simplified as a “zero friction” market.

### Constraint condition

####  Energy balance constraints

10 $$P_{{{\text{pv}}}} + P_{{{\text{grid}}}} + P_{{{\text{fc}}}} = P_{{{\text{el}}}} + P_{{{\text{Eload}}}} + P_{{{\text{EVA}}}} + P_{{{\text{bat}}}}$$

11 $$\begin{array}{*{20}c} {P_{{{\text{gb}}}} + P_{{{\text{fcQ}}}} = P_{{{\text{Qload}}}} } \\ \end{array}$$

12 $$\begin{array}{*{20}c} {\mathop \sum \limits_{{{\text{t}} = 1}}^{{\text{T}}} P_{{{\text{H}}2{\text{load}}}} \left( t \right) \times 3600s = E_{{{\text{H}}2{\text{sum}}}} } \\ \end{array}$$

Formulas (10)–(12) are the balance constraints of electric power, thermal power and hydrogen power respectively. ${\text{P}}_{{{\text{grid}}}}$ is the electric power interacting with the power grid. When it is positive, it means to purchase electric energy from the power grid, and when it is negative, it means to transmit electric energy to the power grid; $P_{{{\text{Eload}}}}$ is the electrical load power, $P_{{{\text{fcQ}}}}$ is the thermal power generated by the fuel cell, and $P_{{{\text{Qload}}}}$ is the thermal load power. $E_{{{\text{H}}2{\text{sum}}}}$ is the total hydrogen load required per day. $P_{{{\text{H}}2{\text{load}}}}$ releases hydrogen energy to supply hydrogen load from hydrogen storage tank.

####  Storage battery restraints

13 $$\begin{array}{*{20}c} { - P_{{{\text{batN}}}} \le P_{{{\text{bat}}}} \left( {\text{t}} \right) \le P_{{{\text{batN}}}} } \\ \end{array}$$

14 $$SOC\left( t \right) = SOC\left( {t - 1} \right) + \frac{{P_{{{\text{batcha}}}} \left( t \right)\eta { }_{{{\text{batcha}}}} }}{{W_{{{\text{bat}}}} }} - \frac{{\frac{{P_{{{\text{batdis}}}} \left( t \right)}}{{\eta_{{{\text{batdis}}}} \left( t \right)}}}}{{W_{{{\text{bat}}}} }}$$

15 $$\begin{array}{*{20}c} {{\text{SOC}}_{{{\text{min}}}} \le SOC\left( t \right) \le {\text{SOC}}_{{{\text{max}}}} } \\ \end{array}$$

where, $P_{{{\text{batN}}}}$ is the rated power of the storage battery; ${\text{SOC }}$ is the state of charge of the storage battery, which is the ratio of the current storage battery capacity to the storage battery capacity; $P_{{{\text{batcha}}}}$ and $P_{{{\text{batdis}}}}$ are the charging and discharging power of the storage battery respectively; $\eta_{{{\text{batcha}}}}$ and $\eta_{{{\text{batdis}}}}$ are the charging and discharging efficiency of the storage battery respectively; $W_{{{\text{bat}}}}$ is the capacity of the storage battery, ${\text{SOC}}_{{{\text{min}}}}$ and ${\text{SOC}}_{{{\text{max}}}}$ are the minimum and maximum values of the state of charge of the storage battery during operation respectively.

####  Hydrogen storage constraints

In order to ensure the safety of the operation process, the operating power of the electrolytic cell and fuel cell should be within a certain range^32,33^, and the hydrogen storage capacity of the hydrogen storage tank has upper and lower limits:

16 $$\begin{array}{*{20}c} {P_{{{\text{elmin}}}} \le P_{{{\text{el}}}} \left( {\text{t}} \right) \le P_{{{\text{elmax}}}} } \\ \end{array}$$

17 $$\begin{array}{*{20}c} {P_{{{\text{fcmin}}}} \le P_{{{\text{fc}}}} \left( {\text{t}} \right) \le P_{{{\text{fcmax}}}} } \\ \end{array}$$

18 $$SOHC\left( t \right) = SOHC\left( {t - 1} \right) + \frac{{P_{{{\text{el}}}} \left( t \right)\eta { }_{{{\text{el}}}} \eta { }_{{{\text{H}}2{\text{in}}}} }}{{W_{{\text{H}}} }} - \frac{{\frac{{P_{{{\text{fc}}}} \left( t \right)\eta { }_{{{\text{fc}}}} }}{{\eta { }_{{{\text{H}}2{\text{out}}}} }}}}{{W_{{\text{H}}} }} - \frac{{\frac{{P_{{{\text{H}}2{\text{load}}}} \left( {\text{t}} \right)}}{{\eta { }_{{{\text{H}}2{\text{out}}}} }}}}{{W_{{\text{H}}} }}$$

19 $$\begin{array}{*{20}c} {{\text{SHOC}}_{{{\text{min}}}} \le SHOC\left( t \right) \le {\text{SHOC}}_{{{\text{max}}}} } \\ \end{array}$$

where, $P_{{{\text{elmin}}}}$, $P_{{{\text{elmax}}}}$, $P_{{{\text{fcmin}}}}$ and $P_{{{\text{fcmax}}}}$ are the minimum and maximum operating power of the electrolytic cell and fuel cell respectively; ${\text{SOHC }}$ is the concept equivalent to the state of charge in hydrogen storage, which is the ratio of hydrogen energy stored in the hydrogen storage tank to the capacity of the hydrogen storage tank^34^. There, $\eta_{{{\text{el}}}}$ and $\eta_{{{\text{fc}}}}$ are the efficiency of the electrolytic cell and the electrical efficiency of the fuel cell, respectively, and $\eta_{{{\text{H}}2{\text{in}}}}$ and $\eta_{H2out}$ are the efficiency of hydrogen storage and hydrogen release in the hydrogen storage tank; $W_{{\text{H }}}$ is the capacity of hydrogen storage tank; ${\text{SOHC}}_{{{\text{min}}}}$ and ${\text{SOHC}}_{{{\text{max}}}}$ are the minimum and maximum values of hydrogen storage state during the operation of hydrogen storage tank.

####  Power constraints of gas-fired boiler

To ensure the safety of the operation process, the operating power of the gas-fired boiler should be within a certain range:

20 $$\begin{array}{*{20}c} {P_{{{\text{gbmin}}}} \le P_{{{\text{gb}}}} \le P_{{{\text{gbmax}}}} } \\ \end{array}$$

where, $P_{{{\text{gbmin}}}}$ and $P_{{{\text{gbmax}}}}$ are the minimum and maximum operating power of gas-fired boiler respectively.

## Simulation example

In order to verify the effectiveness of the economic dispatch strategy proposed in this paper, which considers the carbon trading mechanism and the load optimization of electric vehicles, four comparison schemes are set up for example simulation. The parameters in the carbon trading mechanism are referred to literature^28^. The equipment parameters and source load parameters in the integrated energy system are listed in the appendix. The simulation time is one day (24 hours), and the simulation time step is one hour. The economic dispatch model of the integrated energy system constructed in this paper is a mixed integer linear model, which is solved by using the Yalmip toolkit in MATLAB to call the CPLEX solver .

### Comparison of carbon emissions and total operating costs under four operating schemes.

In view of the introduction of carbon trading mechanism and electric vehicle charging load adjustment strategy proposed in this paper, four comparison schemes are proposed for simulation analysis, and the four schemes are compared and analyzed from the perspective of economic and environmental protection of the introduction of carbon trading mechanism and the positive impact of electric vehicle scheduling, and the relative advantages of the proposed schemes are discussed from the perspective of sustainable development. The operating costs of the four schemes is shown in Table 1.

**Table 1:**
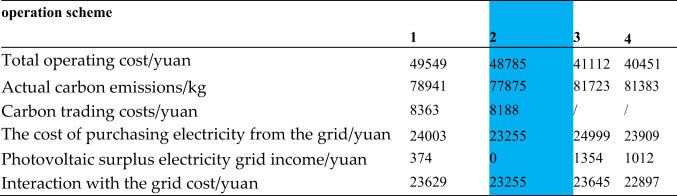
The operating costs of the four schemes.

The blue part in the table shows the simulation results of scenario 2 proposed in this paper.

Option 1: Considering the system carbon trading mechanism, electric vehicles are charged in disorder.

Option 2: Consider the system carbon trading mechanism and adjust the load of electric vehicles.

Option 3: Irregular charging of electric vehicles without considering the system carbon trading mechanism.

Option 4: Electric vehicle load adjustment without considering the system carbon trading mechanism.

#### Economic and environmental protection of introducing carbon trading mechanism

In terms of economy, when not consider the carbon trading mechanism and considers the time-to-use electricity price the total operating cost of scheme 4 which guide the charging of electric vehicles, is the lowest. The daily operation cost of Scheme 2 considering the carbon trading mechanism is 17.08% higher than that of Scheme 4. The main part of the cost increase is the carbon trading cost.

In terms of environmental protection, the actual carbon emission of Scheme 2 is 3508 kg lower than that of Scheme 4, which is 4.5% lower. This shows that considering the carbon trading mechanism will reduce carbon emissions and have better environmental protection.

#### Positive impact of electric vehicle load dispatching

Considering the time-to-use electricity price to guide the charging of electric vehicles, Scheme 2 reduces the total operating cost by 1.54%, carbon emissions by 1.35% and carbon trading costs by 2.09% compared with Scheme 1 of disordered charging, which has more advantages in economy and environmental protection. The load dispatching of electric vehicles reduces the amount of photovoltaic residual electricity on the grid, promotes the consumption of renewable energy, and reduces the power purchase and carbon emissions of the grid. In addition, the disordered charging of electric vehicles is easy to cause random load peaks, which has adverse effects on the stability of the power grid. The problem of large fluctuations in the overall equivalent load in the integrated energy system can be alleviated through the time-of-use electricity price to guide the load curve optimization.

####  Sustainable development

The conservation and maintenance of the environment is conducive to sustainable development and is more important than temporary economic development. With the continuous expansion of the application scale of clean energy, the cost will fall, and there is room for continuous improvement in economy. Compared with the four schemes, only the photovoltaic residual power of Scheme 2 (taking into account the carbon trading mechanism and time-of-use electricity price to guide the charging of electric vehicles) is used for load power supply or energy storage charging, which can fully absorb the output of renewable energy, without the need for grid access, and maintain the stability of the grid. To sum up, Scheme 2 is more in line with the future low-carbon development trend.

### Analysis of typical daily simulation results

Further analyze the day ahead scheduling simulation results of Scheme II (taking into account the carbon trading mechanism and time-sharing electricity price to guide the charging of electric vehicles), discuss from four aspects: power allocation characteristics and reasons, comparison of carbon emissions from electric heating, comparison of various operating costs, and electric vehicle load curve before and after adjustment, and explore the reasons for the simulation results and the positive impact of Scheme II strategy on economy, environment, energy conservation and emission reduction.

####  Power allocation of typical daily simulation results

**Fig. 2:**
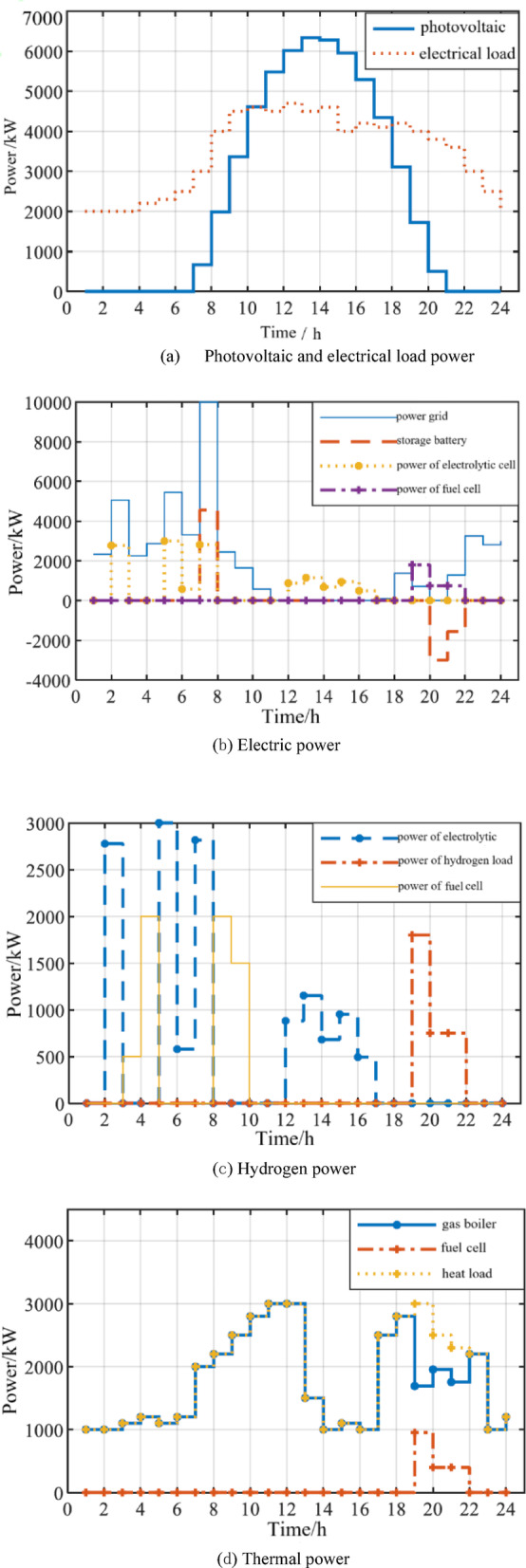
Power distribution of the integrated energy system.

Figure 2a shows the circumstances of the photovoltaic power generation and the electric load power of the industrial park. At 11:00–18:00, there is photovoltaic surplus power, which can be used for energy storage or online; In the rest of the period, PV is not enough to supply the load, and the power shortage needs to be compensated by the grid or energy storage. In Fig. 2b, the electricity power distribution shows that the photovoltaic surplus energy from 12:00 to17:00 provides electricity for hydrogen production in the electrolytic cell, which can reduce the power purchase cost of the grid during the peak power period and thus reduce the total operating cost. According to the latest policy of the National Development and Reform Commission in 2022, the photovoltaic on-grid electricity price is adjusted to the local coal base price. Therefore, for areas with large difference between peak and valley electricity prices, the use of energy storage to absorb the photovoltaic output for peak-valley adjustment is more efficient than the photovoltaic surplus electricity on-grid, and the photovoltaic surplus electricity is preferentially absorbed by the energy storage. The storage battery and hydrogen storage energy are charged during the valley power period and discharged during the peak power period, which can effectively take advantage of the difference between the peak and valley electricity prices to gain profits. It can be seen that the storage battery and fuel cell are in the discharge state at 20:00–21:00, and the electricity price is at the peak. The power purchase of the grid is concentrated in the valley power period to supply the power load when the photovoltaic output is insufficient, and to charge the battery and hydrogen energy storage equipment at the same time.

In Fig. 2c, in the hydrogen power distribution, the hydrogen energy storage system consisting of an electrolytic cell, hydrogen storage tank, and fuel cell first needs to ensure the daily supply of hydrogen load. The electrolytic cell is mainly operated in the valley power period and the period with high PV output, thus reducing the energy purchase cost of the power grid. The output of fuel cells during the peak period of electricity price can improve the comprehensive efficiency of hydrogen energy storage and the overall efficiency of the system; In Fig. 2d, in thermal power distribution, fuel cell cogeneration supplies part of the thermal load, reducing the cost of purchasing natural gas and the carbon emissions generated by gas fired boilers.

####  Comparison of carbon trading volume of gas-fired boiler and power grid in integrated energy system

**Fig. 3:**
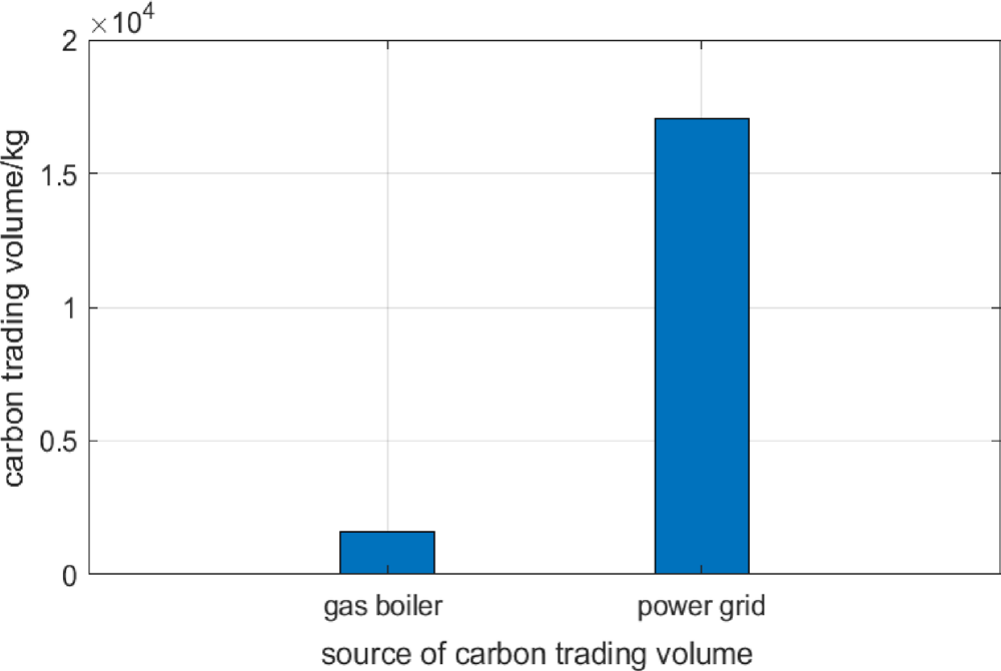
Gas boiler, power grid purchase carbon trading volume of integrated energy system.

From the perspective of the proportion of environmental costs, the carbon trading costs generated by electricity purchase are the main. In Fig. 3, on the one hand, because the electricity load is large, the electricity purchased from the grid is large, and the actual carbon emissions are high; On the other hand, because the carbon emission coefficient of power generation units is larger than that of heat generation units, the carbon trading volume and carbon trading cost of power purchase are higher for the same load.

####  Comparison of daily operation costs

**Fig. 4:**
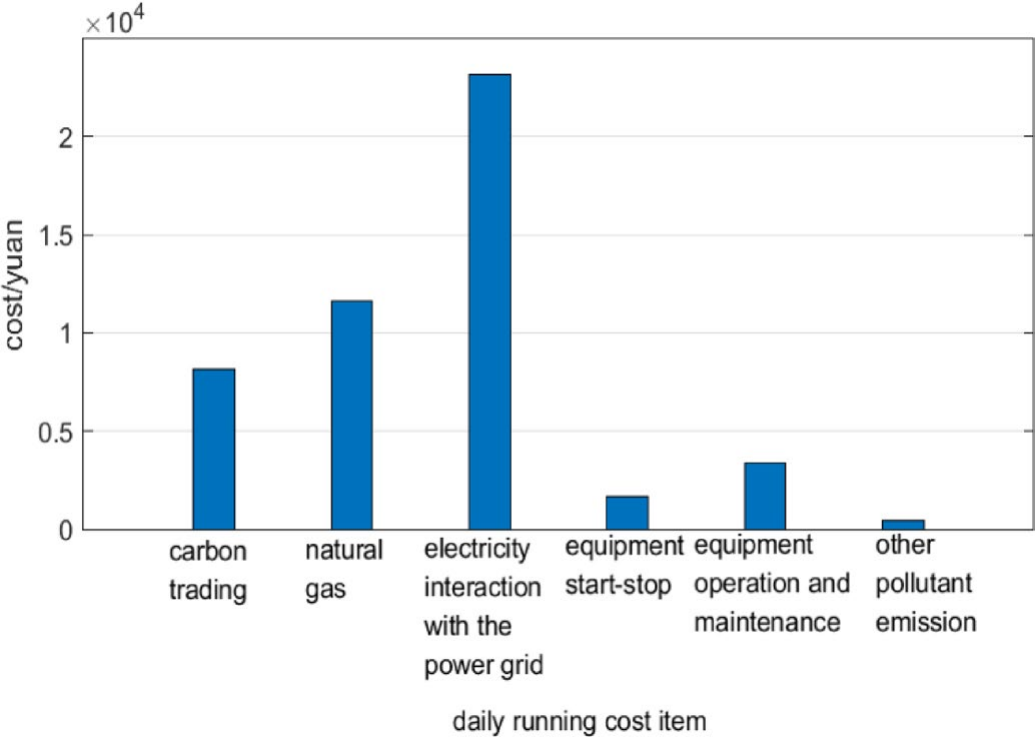
Various costs of integrated energy system.

The Various costs of integrated energy system is shown in Fig. 4. In general, the total operation cost is dominated by the cost of power grid energy exchange and the cost of purchasing natural gas, accounting for 71.53% of the total cost, which is consistent with the main function of the system to supply electricity, heat and hydrogen loads; The carbon transaction cost accounts for 16.78% of the total cost, second only to the energy purchase cost, indicating that the system has a strong punishment on carbon emissions and can play a greater role in reducing carbon emissions; The cost of equipment startup, operation and maintenance and the environmental cost of other pollutants are relatively small, accounting for 11.68% of the total cost.

####  Electric vehicle load curve before and after optimization

**Fig. 5:**
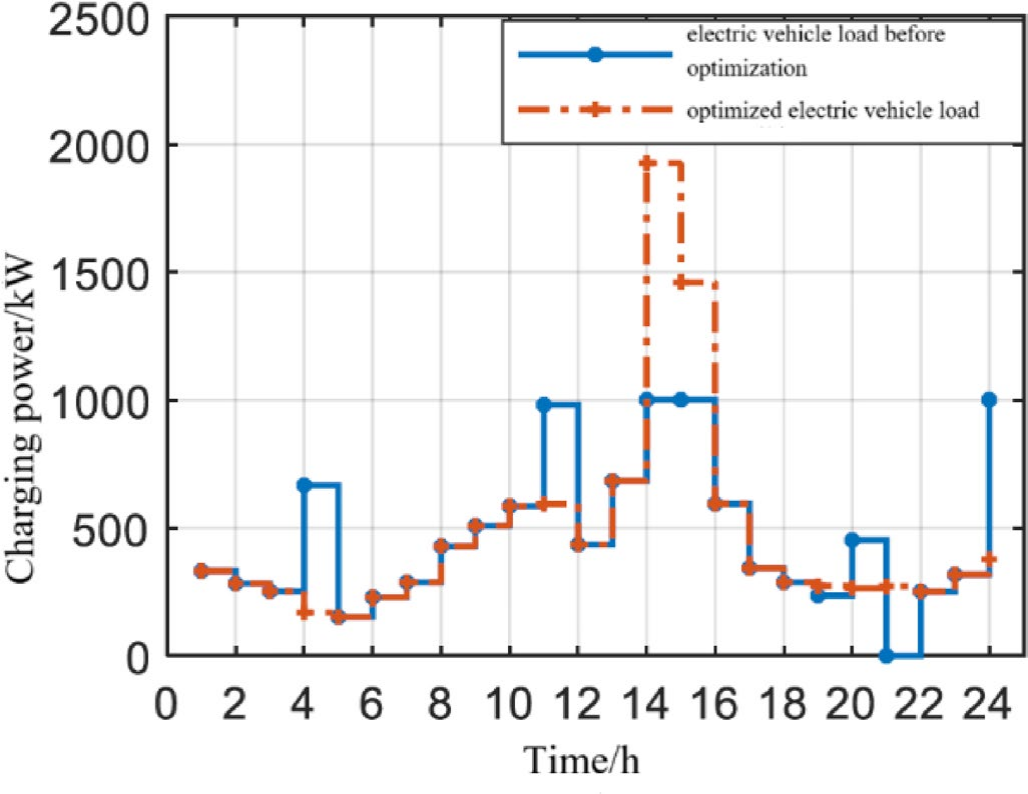
Electric vehicle load curve.

It can be seen from Fig. 5 that the load curve of electric vehicles before optimization fluctuates greatly, especially at 14:00, the load peak appeared, and the peak-to-valley difference of daily load reached 1774kW, which is not conducive to the internal stability of the integrated energy system, and in the peak-to-valley electricity price period, it is disorderly charging, with high charging cost and poor economy. After optimizing the electric vehicle load as the internal load of the integrated energy system, it first significantly reduces the peak to valley load difference, which is conducive to system stability; Secondly, on the premise that the charging load adjustment ratio is not greater than 0.3, the load during some peak power periods is moved to valley power and flat power periods, improving economic efficiency. It is at the peak electricity price period, but the charging load after optimization is higher than that before optimization at 11:00 am. This is because at this time, the photovoltaic output is much greater than the basic load. Using photovoltaic surplus power to power electric vehicles is more economical than power grid power supply.

## Sensitivity analysis

### Sensitivity analysis of carbon trading mechanism

The operating costs of the four schemes is shown in Table 2. Comparing the carbon trading base price of 0.152 yuan and 0.252 yuan, it was found that the higher the carbon trading base price, the lower the carbon trading cost directly, but had no impact on carbon emissions; Compared to the carbon trading base price of 0.5 yuan and 0.252 yuan, the carbon trading cost increased significantly, increasing by 94.31%, and the actual carbon emissions decreased by 0.87%; Compared to the carbon trading base price of 0.748 yuan and 0.5 yuan, the carbon trading cost increased, but the actual carbon emissions did not decrease. This indicates that as the carbon trading base price increases, the higher the carbon trading cost, which has a certain promoting effect on the reduction of actual carbon emissions. When the carbon trading base price rises to a certain value, increasing the carbon trading base price will no longer have a significant effect on the reduction of carbon emissions.

**Table 2:**
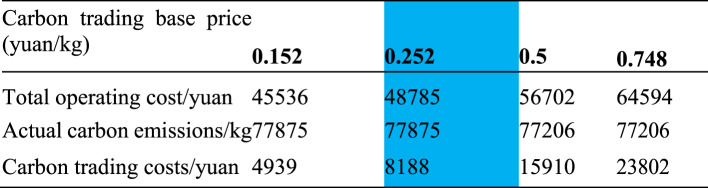
The operating costs of the four schemes.

The blue part in the table shows the simulation results of scenario 2 proposed in this paper.

### Effect of adjustable proportion of electric vehicle charging load

Simulation results of different charging load adjustment ratio is shown in Table 3. When the adjustable proportion of electric vehicle load is not greater than 0.6, 0.5, or 0.4, the actual carbon emissions remain unchanged, indicating that changes in the electric vehicle load curve do not affect the purchase of electricity from the grid. However, the cost of interaction with the power grid is different, that is, the amount of photovoltaic power on the grid is different. As the adjustable proportion of electric vehicle load decreases, the cost of interaction with the power grid increases, indicating that the amount of photovoltaic power on the grid decreases, that is, photovoltaic excess power is more used to charge electric vehicles than to connect them to the grid; The total charging cost of electric vehicles shows an increasing trend as the adjustable proportion of electric vehicle load decreases. For each 0.1 decrease in the adjustable proportion of electric vehicle load, the total charging cost growth rates are 3.39% and 3.28%, respectively. When the adjustable load ratio of electric vehicles is not higher than 0.3, the total charging cost increases by 13.09% compared to 0.6, but the cost of interaction with the power grid and the cost of purchasing natural gas have decreased slightly; From the perspective of total operating cost, every 0.1 decrease in the adjustable proportion of electric vehicle load will result in a total operating cost growth rate of 0.59%, 0.59%, and 1.15%,Considering the urgency of charging time for electric vehicles in practical situations, and given the small increase in total operating costs, it is considered reasonable to limit the adjustable load ratio of electric vehicles to no more than 0.3.

**Table 3:**
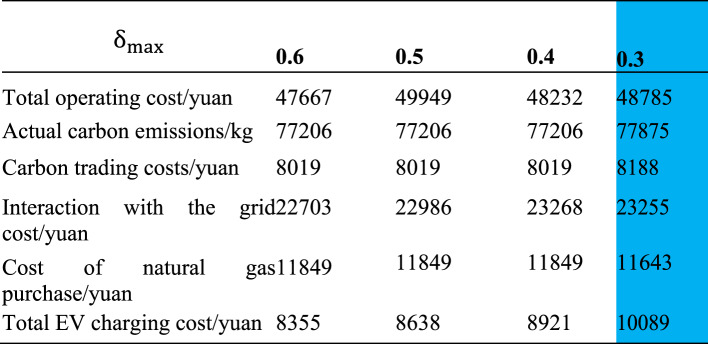
Simulation results of different charging load adjustment ratio.

The blue part in the table shows the simulation results of scenario 2 proposed in this paper.

From the electric vehicle load curves before and after optimization under different adjustable proportion constraints of electric vehicle load, it can be seen that the higher the adjustable proportion of electric vehicle load, the more the load moves from peak power period to valley power period, that is, the more obvious the adjustment of electric vehicle load.

### Impact of time-of-use electricity price on operation cost and dispatching results

**Fig. 6:**
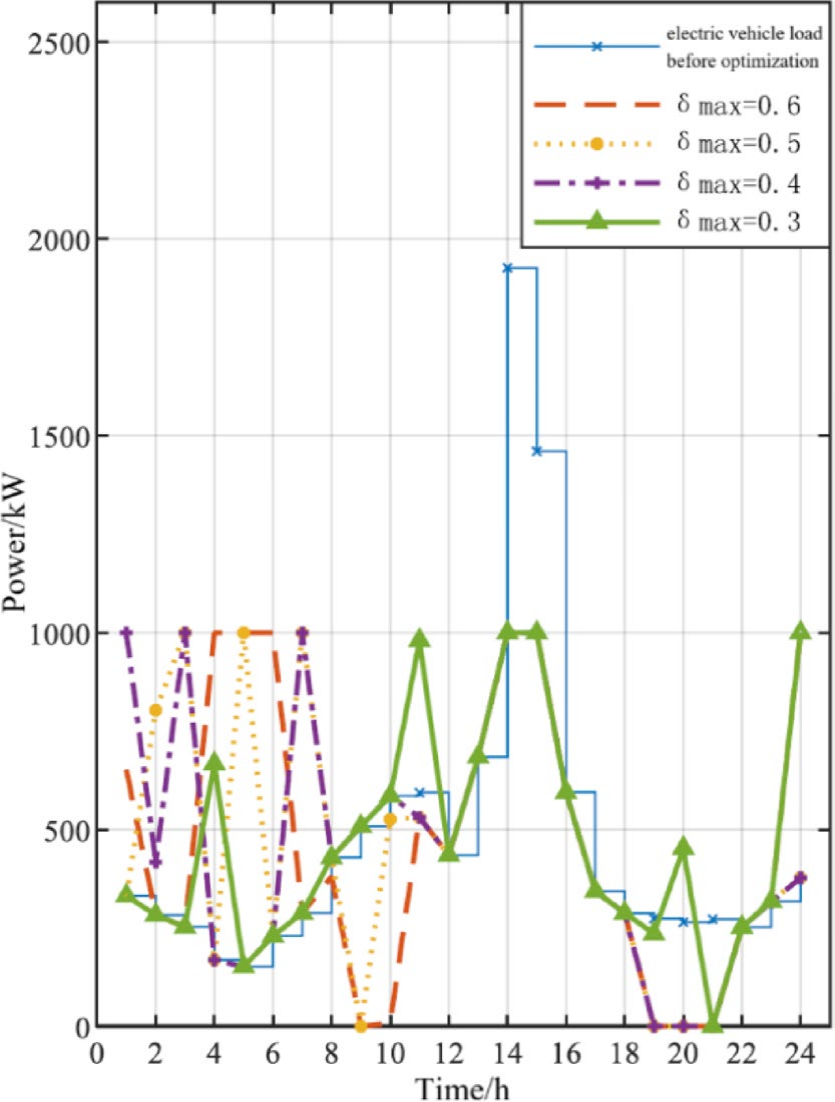
Load curves of electric vehicles corresponding to different charging load adjustment ratio.

The time-to-use electricity price mentioned above is based on the time-to-use electricity price of the industrial and commercial industries in Beijing. For different regions, the time of use electricity price varies. This section takes Xi’an’s time-to-use electricity price as an example and compares it with Beijing’s time-to-use electricity price to study the impact of time-to-use electricity price on various operating costs, power distribution of various parts, and optimization of electric vehicle load curve.The load curves of electric vehicles corresponding to different charging load adjustment ratio is shown in Fig. 6.

In Table 4, the overall time-of-use electricity price in Xi’an is lower than that in Beijing, with short peak power periods and small peak valley electricity price differences. Therefore, overall, the total operating cost is lower than the total operating cost under the time-to-use electricity price in Beijing. The benefits of photovoltaic surplus power grid access under the time-to-use electricity price in Xi’an indicate that when the peak and valley electricity price difference is small, the benefits of partial surplus power grid access for renewable energy are better than those of full charging of stored energy.

**Table 4:** Comparison of various costs with different time-to-use prices.

Time-of-use price cities	Xi’an	Beijing
Total operating cost/yuan	47,073	48,785
Actual carbon emissions/kg	77,563	44,875
Carbon trading costs/yuan	7945	8188
Cost of electricity purchase	23,188	23,255
Photovoltaic surplus electricity grid revenue	654	0

**Fig. 7:**
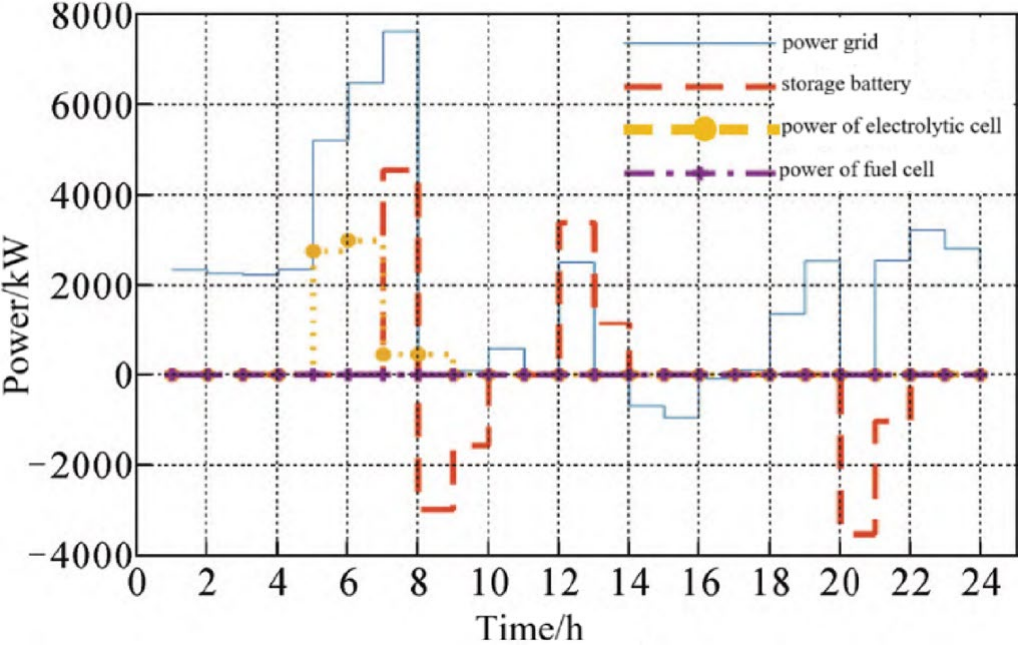
Charging and discharging output of electric vehicles in Xi’an city.

As can be seen from the Fig. 7, when there is surplus power from the photovoltaic output, only a small portion of the electricity can be used to charge the battery, while most of the electricity is online. However, hydrogen energy storage can generate significant losses during the energy conversion process, resulting in low electrical efficiency. If it cannot convert renewable energy output, hydrogen energy storage is less economical.

**Fig. 8:**
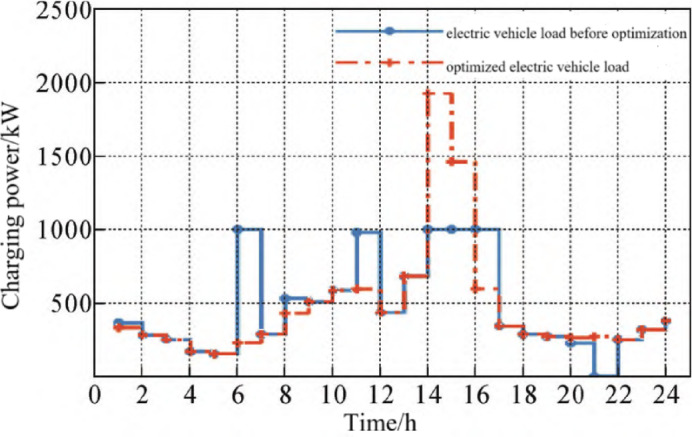
Load curve of electric vehicles with time-of-use price in Xi’an city.

In Fig. 8, the overall trend of electric vehicle power curves before and after optimization guided by time-to-use electricity prices in both cities is similar, but due to the lower electricity prices in Xi’an, the overall charging cost is also lower.

## Conclusion

An integrated energy system suitable for the industrial park is constructed. The hydrogen energy chain has been added on the basis of the electric and thermal energy structure of the traditional integrated energy system based on the load characteristics of the industrial park. The carbon trading mechanism and the time-sharing electricity price in the park has been considered to optimize the electric vehicle load curve. The CPLEX solver has been used to calculate the day ahead scheduling results with the minimum daily operating cost. The specific conclusions are summarized as follows:

1. Having considered the demand for hydrogen energy in the industrial park, a hydrogen energy chain has been added to the comprehensive energy system to achieve a combined energy supply mode of hydrogen energy supply and fuel cell cogeneration.
2. Having taken the charging load of electric vehicles in the park as an adjustable load, the daily operating cost can reduced by 1.54% when having adjusted the load ratio of electric vehicles below 30%. The absorption of photovoltaic output can be significantly promoted by optimizing the load curve of electric vehicles.
3. The carbon emissions can be reduced by 4.5% during daily operation based on the stepped carbon trading mechanism. It has been proved that the introduction of carbon trading mechanisms and electric vehicle load regulation models can have the greatest comprehensive advantages in reducing carbon emissions, improving economy, and absorbing renewable energy output.
4. The optimization results under different time-to-use electricity prices in Xi’an city and Beijing city are compared. The higher the peak valley electricity price difference, the more conducive it is to absorb renewable energy output and improve energy storage and utilization.

## Data Availability

The datasets used and/or analysed during the current study are available from the corresponding author on reasonable request.
